# Management of Long-COVID Postural Orthostatic Tachycardia Syndrome With Enhanced External Counterpulsation

**DOI:** 10.7759/cureus.18398

**Published:** 2021-09-30

**Authors:** Swathi Varanasi, Mohankrishnan Sathyamoorthy, Sanjayanth Chamakura, Sachin A Shah

**Affiliations:** 1 Medical Affairs, Flow Therapy, Fort Worth, USA; 2 Internal Medicine, University of North Texas Health Science Center, Fort Worth, USA; 3 Cardiology, Medical City North Hills, North Richland Hills, USA; 4 Pharmacy, University of the Pacific, Stockton, USA

**Keywords:** postural orthostatic tachycardia syndrome, covid long haul syndrome, eecp, pasc, long covid

## Abstract

A growing number of patients diagnosed with COVID-19 disease have been reported to have postural orthostatic tachycardia syndrome (POTS) after the acute phase. A 57-year-old female was diagnosed with COVID-19 in December 2020. As a result of her acute illness, she was hospitalized for COVID pneumonia and respiratory failure, followed by stays at an acute care facility and home rehabilitation center. After the acute phase, the patient was diagnosed with long-COVID-19-associated POTS with symptoms such as fatigue, “brain fog,” and dyspnea. The patient was referred to an enhanced external counterpulsation (EECP) treatment center and underwent 15, one-hour sessions over three weeks. Upon completion of therapy, the patient reported improvements with “brain fog” and the ability to perform activities of daily living. Her Patient-Reported Outcome Measurement Information System (PROMIS) Fatigue score was reduced by three points, six-minute walk distance increased by 85 feet, and Duke Activity Status Index (DASI) improved by over 15 points. EECP therapy was chosen due to the overlap in underlying pathology driving POTS and the mechanisms of action of EECP. This report is the first case of using EECP for the successful management of COVID-19-associated POTS and warrants further trials.

## Introduction

COVID-19 is a global pandemic with significant post-acute sequelae, also referred to as long-COVID or post-acute sequelae of COVID-19 (PASC) [1]. While over 50 long-COVID symptoms have been documented, symptoms with higher than 20% prevalence include fatigue, headache, attention disorder or impaired cognitive function (“brain fog”), hair loss, dyspnea, ageusia, anomia, and post-activity polypnea. Of the patients with long-COVID, 80% experienced more than one symptom [2]. Orthostatic tachycardia is often paired with these common symptoms, resulting in a diagnosis of post-COVID-19 postural orthostatic tachycardia syndrome (POTS) defined as excessive orthostatic tachycardia (increase of >30 bpm for adults and >40 for 12-19 years old) within 10 minutes of transitioning to an upright or standing posture without orthostatic hypotension for three months.

Several case reports documenting patients experiencing long-COVID POTS have been recently published, with some estimates suggesting that 25%-50% of patients report tachycardia or palpitations for at least 12 weeks [3-5]. The American Autonomic Society has issued a statement for the need for more resources and research for the appropriate management of post-COVID POTS, but no consensus approaches to treatment exist. Enhanced external counterpulsation (EECP) is a noninvasive therapy that is approved by the Food and Drug Administration for its use in chronic stable angina and ischemic heart failure. This therapy involves 35 one-hour sessions over the course of seven weeks. Patients lie on a treatment bed with blood pressure-like cuffs that inflate and deflate in accordance with the cardiac cycle. Typically, candidates for EECP include those with chronic stable angina (Canadian Cardiovascular Society (CCS) angina class III or IV) and heart failure with ischemic etiology. Due to the unique mechanism of action of EECP, benefits have been seen in other conditions such as diabetes, hypertension, and erectile dysfunction.

We postulated that EECP may have a treatment benefit in long-COVID-associated POTS due to its ability to improve dysautonomia and chronic inflammatory responses [6]. We present a patient case of long-COVID-associated POTS successfully managed with EECP therapy.

## Case presentation

A 57-year-old White female with a history of coronary artery disease (CAD) and disabling angina (CCS III-IV) was diagnosed with COVID-19 in December 2020. Her past medical history included hypertension, dyslipidemia, anxiety, attention-deficit/hyperactivity disorder (ADHD), anemia, neuropathy, bilateral foot drop, osteoarthritis, Ehlers-Danlos syndrome, Budd-Chiari syndrome, and chronic pain. She was hospitalized due to COVID pneumonia and respiratory failure for seven weeks. During her hospital stay, she was intubated and experienced complications including retroperitoneal bleeding and bilateral deep vein thrombosis. She subsequently spent three weeks at a long-term acute care facility, followed by three weeks at a rehabilitation center. 

After returning home, she required oxygen (2 L/day) therapy for her chronic respiratory failure with her lung capacity being at 35%-40%. She sustained a chronic cough along with an aching chest, fatigue, “brain fog,” shortness of breath (SOB), and dyspnea upon exertion (DOE). She reported orthostatic symptoms when ambulating and needed to sit down for up to 10 minutes to recover to baseline. The exact POTS diagnosis date is unknown. Five months after her acute phase, the patient was referred for her symptoms to an EECP treatment center for a modified abbreviated regimen of 15, one-hour treatment sessions (three times a week for five weeks) based on previous data [7]. Her pretreatment blood pressure on day one of EECP treatment was 95/67 mmHg and posttreatment blood pressure upon completion of therapy on day 15 was 105/57 mmHg.

After nine, one-hour treatments of EECP over three weeks, the patient reported an improvement in chest pain, SOB/DOE, and fatigue. She indicated that although she continued to experience symptoms, they were less frequent and less severe and occur for a shorter duration. 

Upon completion of 15 sessions, the patient reported having less “brain fog” and feeling “amazing,” with the ability to perform physical and mental tasks she was incapable of prior to EECP. The patient reported that she was using her cane less and was able to walk from one side of the grocery store to the other, which she was unable to do before initiating EECP. This was in line with her six-minute walk test distance improving from 910 ft at baseline to 995 ft post-EECP. Her Patient-Reported Outcome Measurement Information System (PROMIS) Fatigue score decreased from moderate (score = 15) to mild (score = 12) fatigue. Her CCS class decreased by one, and her Duke Activity Status Index (DASI) score improved from 0 to 15.45. Her Seattle Angina Questionnaire and Rose Dyspnea Scale scores remained relatively unchanged.

## Discussion

Emerging data suggest a link between POTS and COVID-19. The mechanism of post-COVID POTS remains unknown, although inflammatory and autoimmune responses, including the generation of autoantibodies, may be at play [6]. It is postulated that the cytokine storm from the COVID-19 infection results in sympathetic dysregulation, causing the abnormal autonomic response in POTS [6]. When transitioning to sitting up or standing, patients with POTS become tachycardic due to the narrowing of blood vessels, hindering cerebral and coronary blood flow. The release of epinephrine and norepinephrine in orthostatic imbalance likely drives some long-COVID symptoms such as palpitations and shortness of breath.

In comparison with the general population, patients with POTS have an increased prevalence of conditions such as Hashimoto’s thyroiditis, rheumatoid arthritis, and celiac disease, suggesting autoimmune activity [4]. Most patients with POTS have elevated autoantibodies to at least one of a wide range of receptors associated with autonomic function: adrenergic receptors (beta1 and beta2), muscarinic receptors (M2 and M4), acetylcholine receptors (N type and P/Q type), opioid-like 1 receptors, and angiotensin II type 1 receptors [8]. These biomarkers in patients with POTS reinforce the notion that there is an autoimmune component to its pathophysiology. These autoantibodies, along with cardiovascular and neuronal function, support that the relationship between COVID-19 and POTS is attributed to autonomic dysregulation. More studies are needed to determine the precise mechanism with which both COVID-19 and POTS are interconnected through the autonomic nervous system.

Based on a study by Chopoorian et al., patients with POTS had reduced flow-mediated dilation when compared to healthy controls, suggesting that POTS is characterized by underlying endothelial dysfunction [9]. Interestingly, EECP has demonstrated beneficial effects on peripheral artery flow-mediated dilation and endothelial-derived vasoactive agents in patients with symptomatic coronary artery disease [10]. The mechanism behind EECP involves a retrograde aortic flow that significantly decreases the number of pro-inflammatory cytokines (e.g., tumor necrosis factor-alpha, monocyte chemoattractant protein-1, soluble vascular adhesion molecule-1, high-sensitivity C-reactive protein, and lipid peroxidation marker 8-isoprostane) and increases growth factors (e.g., vascular endothelial growth factor-1). EECP is also known to increase plasma nitric oxide metabolites (nitrate and nitrite (NOx)) and 6-keto-prostaglandin F1α (6-keto-PGF1α) and decrease endothelin-1 (ET-1), impacting overall hemodynamics [10]. Undergoing EECP therapy can enhance coronary blood flow, increase cardiac efficiency, and facilitate angiogenesis - all producing physiological benefits in POTS. Of note, EECP is contraindicated in patients who are pregnant; have active thrombophlebitis, bleeding diathesis, or severe lower extremity vaso-occlusive disease; or with a large (>5 mm) or an unstable abdominal aortic aneurysm.

POTS is usually managed with a combination of exercise, lifestyle, and medical management. The common protocols for the management of POTS symptoms include exercises in the recumbent position, such as physical counterpressure techniques, to help stabilize blood pressure and heart rate. Some of the nonpharmacological interventions proven successful in patients with POTS include avoidance of precipitating factors, hydration, compression stockings, sleeping with the head elevated, and resistance exercises [11]. Currently, no drugs have been shown to reverse long-COVID or long-COVID POTS in controlled or large-scale cohort studies.

## Conclusions

In this report, we describe the first case we are aware of using EECP for the management of long-COVID-associated POTS. The well-established physiological mechanisms of action of EECP and the positive findings from this case suggest that EECP may be a suitable treatment modality for long-COVID symptoms. We view it important to devote greater resources to evaluate the use of EECP in COVID-19-associated and standard POTS.
